# Application and Evaluation of Highly Automated Software for Comprehensive Stent Analysis in Intravascular Optical Coherence Tomography

**DOI:** 10.1038/s41598-020-59212-y

**Published:** 2020-02-07

**Authors:** Hong Lu, Juhwan Lee, Martin Jakl, Zhao Wang, Pavel Cervinka, Hiram G. Bezerra, David L. Wilson

**Affiliations:** 1 Present Address: Microsoft, Azure Global, Cambridge, MA 02142 USA; 2 Department of Biomedical Engineering, Case Western Reserve University, Cleveland, OH 44106 USA; 3 First Department of Cardio-Angiology and Internal Medicine, Faculty Hospital Hradec Kralove, Hradec Kralove, Czech Republic; 4 Department of Electrical Engineering and Computer Science, Research Laboratory of Electronics, Massachusetts Institute of Technology, Cambridge, MA 02139 USA; 5 Department of Cardiology, Krajska zdravotni a.s., Masaryk Hospital, UJEP Usti nad Labem, Usti nad Labem, Czech Republic; 6 Cardiovascular Imaging Core Laboratory, Harrington Heart & Vascular Institute, University Hospitals Cleveland Medical Center, Cleveland, OH 44106 USA; 7 Department of Radiology, Case Western Reserve University, Cleveland, OH 44106 USA

**Keywords:** Interventional cardiology, Biomedical engineering

## Abstract

Intravascular optical coherence tomography (IVOCT) is used to assess stent tissue coverage and malapposition in stent evaluation trials. We developed the OCT Image Visualization and Analysis Toolkit for Stent (OCTivat-Stent), for highly automated analysis of IVOCT pullbacks. Algorithms automatically detected the guidewire, lumen boundary, and stent struts; determined the presence of tissue coverage for each strut; and estimated the stent contour for comparison of stent and lumen area. Strut-level tissue thickness, tissue coverage area, and malapposition area were automatically quantified. The software was used to analyze 292 stent pullbacks. The concordance-correlation-coefficients of automatically measured stent and lumen areas and independent manual measurements were 0.97 and 0.99, respectively. Eleven percent of struts were missed by the software and some artifacts were miscalled as struts giving 1% false-positive strut detection. Eighty-two percent of uncovered struts and 99% of covered struts were labeled correctly, as compared to manual analysis. Using the highly automated software, analysis was harmonized, leading to a reduction of inter-observer variability by 30%. With software assistance, analysis time for a full stent analysis was reduced to less than 30 minutes. Application of this software to stent evaluation trials should enable faster, more reliable analysis with improved statistical power for comparing designs.

## Introduction

Intravascular optical coherence tomography (IVOCT) has been widely used in clinical trials to assess the efficacy and safety of stent designs. With superior resolution, sensitivity and imaging speed, IVOCT has enabled visualizing deployment of the stent structure and arterial healing following implantation^1–4^. Stent struts are very clearly seen enabling determination of stented area and stent malapposition. It has been shown that IVOCT is more sensitive in identifying thin stent tissue coverage compared to intravascular ultrasound and quantifications using IVOCT are more reproducible^5,6^. Lack of stent strut coverage and apposition has been related to increased risk of stent thrombosis after drug eluting stent implantation^7–9^. Multiple stent trials used strut coverage assessed by IVOCT as their primary end point^10–14^.

Current analysis of IVOCT stent pullbacks is primarily done manually with very limited software assistance. Manual stent analysis is extremely tedious and time consuming. Each stent pullback contains 100~200 stent frames and more than a thousand struts need analyzing, thereby requiring about 6–12 hours for analysis of a full stent. To save time and labor, stent trials usually analyze pullbacks with skipped frames. Analysis time is a limiting factor in the size of stent trials, which in turn limits the ability to obtain statistical significance. In addition, even with careful training and rigorous quality control, there exists inter-observer variability which limits the ability to obtain statistical significance between two stent designs. Thrombosis risk is thought to depend upon the presence of uncovered and malapposed struts. Identification of covered versus uncovered struts can be most challenging when there is very thin tissue coverage, leading to variability among analysts. This makes it difficult to compare stents analyzed at different institutions or to compare new designs to previously analyzed stent types. Obviously, a computerized stent analysis solution is desirable to reduce time and labor cost and improve objectivity and reproducibility of stent analyses.

There are previous publications by ourselves and others partially solving the problem of stent analysis^15–27^. A few algorithms have been proposed to detect lumen boundary and stent struts. However, very little work has been done to accomplish the more challenging and more important task of classifying stent struts as covered versus uncovered. Ughi *et al*. proposed an automated analysis method for measuring distance between the lumen and detected strut^20^. They acquired IVOCT images from rabbit iliac arteries, and showed good correlations of coverage quantification among the automatic analysis, manual analysis, and histological assessment. In addition, they reported that only using distances between struts and lumen boundary is not sufficient for differentiating thinly covered and uncovered struts. Nam *et al*. developed automated stent strut analysis approach using a relatively small number of 20 *in vivo* pullbacks comprising 12 baseline and 8 follow-up cases^26^. Before strut detection, they utilized a series of pre-processing including catheter and guide-wire removal, image shifting, and lumen boundary detection. Then, the strut candidates were pre-determined using first and second gradient information, and classified as either non-strut or strut. These reports underscore the need for additional algorithm development and validation. Most recently, our group proposed the fully-automated stent coverage analysis method to quantitatively evaluate the stent tissue coverage^27^. This method enabled to classify covered and uncovered struts, measure tissue thickness, and determine clusters of uncovered struts. In order to avoid the potential inter-observer variability, we particularly performed active learning relabeling for all pullbacks.

Our group has proposed multiple image analysis methods on IVOCT image volumes, including segmentation and plaque characterization^28–35^. Some approaches have attempted to address each stent analysis step. Lumen boundary and guidewire were efficiently segmented using dynamic programming (DP)^28,29,36^. We devised multiple image features to detect stent struts^25^ and also used a Bayesian network and graph search^37^. Building upon strut detection, we have determined the presence of strut tissue coverage^27^ using advanced machine learning algorithms and automatically measured tissue coverage thickness. The Cardiovascular Imaging Core Laboratory in the Harrington Heart & Vascular Institute, University Hospitals Cleveland Medical Center, Cleveland, Ohio, hereafter called the Core Lab, has a very large database of manually analyzed stents, providing us with a large amount of examples for training robust classifiers and evaluating algorithm performance.

In this report, we demonstrate and evaluate the clinical application of a highly automated, comprehensive software package, OCT Image Visualization and Analysis Toolkit for Stent (OCTivat-Stent), based on the above algorithms with added features for editing and measurement. As image artifacts or poor image quality can compromise algorithm performance, we advocate manual review and potential editing of automatic processing output for more accurate results. We applied OCTivat-Stent to 292 stent pullbacks without tissue coverage (baseline images immediately following implantation) and with tissue coverage (follow up studies). Many of the pullbacks came from the Comparison of Biolimus A9 and Everolimus Drug-Eluting Stents in Patients With ST Segment Elevation Myocardial Infarction (ROBUST) study (NCT00888758). Clinical results of ROBUST are presented elsewhere^38,39^. Automated results were reviewed in the software and edited by cardiologist analysts. To evaluate software usability, the automatic analysis time, manual review and editing time, and numbers of edits were all assessed. To assess accuracy, automated area and tissue thickness measurements were compared to manual assessments obtained using a commercial product. For comparing stent designs, good measurement precision is important to enable powered studies. Strut level analysis is important as it allows one to determine the presence of uncovered struts, which can create a thrombosis risk^40^, but it also creates the greatest challenge for human interpretation. Hence, we compared intra-analyst variability of strut level measurements using OCTivat-stent followed by manual editing to that obtained with a fully manual analysis using a commercial workstation product.

## Materials and Methods

### Data acquisition

IVOCT pullbacks were acquired by a Fourier-Domain OCT (FD-OCT) system (C7-XR OCT Intravascular Imaging System, St. Jude Medical, Westford, MA). The system used a tunable laser light source sweeping from 1250 *nm* to 1370 *nm*, providing 15 *µm* resolution along the A-line and 20~40 *µm* lateral resolution. Pullback speed was 20 *mm/sec* over a distance of 54.2 *mm*, and frame interval was 200 *µm*, giving 271 frames in total. Most of the time, 100 to 200 frames had stents present, depending upon the length of the stent.

We analyzed 103 baseline pullbacks (at the time of implantation) and 189 follow-up pullbacks (≈9 months following implantation), mostly from the ROBUST study. Fifty of the follow-up pullbacks were previously analyzed using a commercial offline analysis software (St. Jude Medical, Westford, MA). In the commercial software, lumen boundaries were automatically traced, manually reviewed, and edited. Struts were manually marked, and the stent contour was automatically created. For the remainder of this paper, we refer to this analysis as “manual analysis” for conciseness. On these 50 pullbacks, we compared multiple area measurements obtained by the highly automated OCTivat-Stent against manual analysis results. On the full dataset, we analyzed the number of manual edits and editing/review time in OCTivat-Stent.

Inter-observer variability is a limitation of stent studies relying on fully manual analysis. To examine how inter-observer variability is affected with software assistance, we asked three analysts to perform manual analysis and OCTivat-Stent analysis, with editing, on three pullbacks. There was a delay greater than two weeks between analyses, to limit any confound of memory from the first analysis. We used every image frame that was considered analyzable by human analysts. We asked expert analysts to mark the start and end frames of each analyzable pullback segment. The images with poor quality were excluded to avoid misleading results.

### Functions of stent analysis software

**Figure 1:**
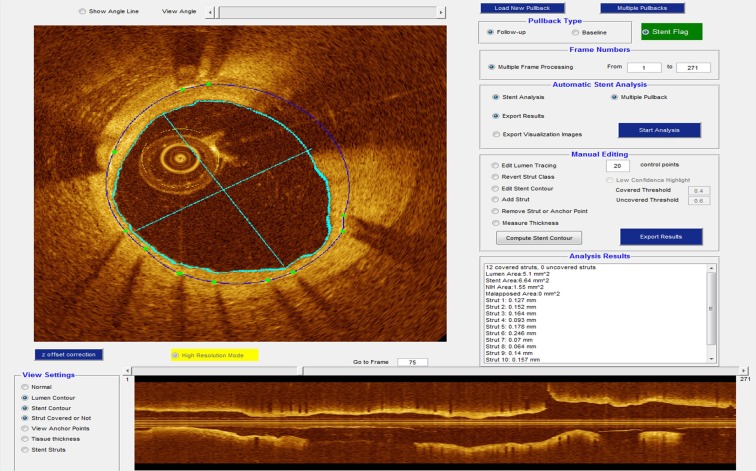
OCTivat-Stent software. This software provides various tools for visualizing and analyzing IVOCT pullback images of stents. See text for details.

Stent analysis with OCTivat-Stent consists of fully automated, offline analysis followed by interactive expert review and potential editing of results (Fig. 1). Automatic stent analysis includes guidewire artifact removal, lumen boundary detection, stent strut detection and classification as covered versus uncovered, stent/lumen/tissue coverage area, and strut level coverage thickness quantification. Automated analysis is initiated following loading a pullback and identifying its start and stop frames for the stent. For convenience, multiple pullbacks can be set up and analyzed in batch mode. User-friendly tools were included in OCTivat-Stent for convenient expert review and editing. Principal editing functions include: 1) Edit lumen tracing. 2) Add missed struts or remove false positive struts. 3) Edit stent contour. 4) Reclassify covered or uncovered struts. 5) Correct strut tissue coverage thickness measurement. Interactive tools were created using Matlab graphical user interface development environment (MathWorks Inc., Natick, MA, USA). Lumen tracing and stent contour editing are done with convenient dragging functions. Adding, removing, and reclassifying struts are done with simple clicks. These functions were optimized through multiple rounds of modification based on user feedback.

After automatic analysis and manual review and editing, the software generates an analysis report including lumen area and diameter, stent area, tissue coverage area, malapposition area, strut location, class (either covered, uncovered, or malapposed), and coverage thickness/malapposition distance. Figure 1 demonstrates an example stent analysis result from IVOCT pullback.

## Image Analysis Algorithms

Below, we briefly review some previously developed algorithms as well as recent improvements that are core functions in OCTivat-Stent.

### Guidewire segmentation

**Figure 2:**
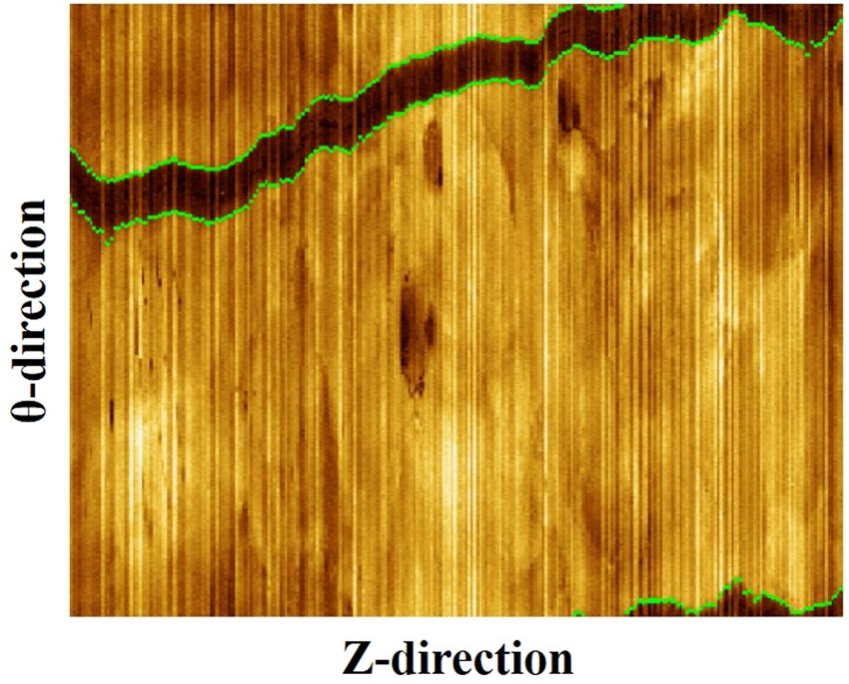
Guidewire detection result (green) in en-face (*θ*, *z)* view. En-face image was created using 271 IVOCT images, where z refers to length along the artery.

In IVOCT images, the guidewire presents as a bright, thick arc followed by a wide shadow due to its high reflectivity. This region must be excluded from analysis as there is no way to visualize the portion of the stent behind the guidewire. To segment the guidewire, averaged A-line values of each frame were collected as a function of frame number to form a 2D, (*θ*, *z*) image, where z refers to length along the artery, in Fig. 2. The guidewire shadow forms a continuous dark band in this image along *z*. An objective function of pixel value difference is applied to the top and bottom boundaries of the dark band, but with different signs. DP was then applied twice to find these two boundaries^28,29^.

### Lumen segmentation

To accurately quantify lumen diameter, tissue coverage area, and malapposition area, a robust lumen segmentation method is needed. Lumen segmentation also serves as a cornerstone for further analysis such as plaque segmentation and stent strut detection. We used a DP, lumen segmentation algorithm^28,29^. Briefly, for each polar coordinate, (*r*, *θ*), image, an intensity gradient map was created by convolving the image with an edge detection kernel along *r*. DP was then applied to this gradient map to find the boundary having the highest cumulative gradient from top to bottom along *θ*.

### Stent strut detection

In order to perform comprehensive stent analysis, a robust stent strut detection method is prerequisite. In^25^, we proposed a machine learning-based algorithm to detect stent struts. Briefly, we first obtained a large number of candidate struts using image processing techniques. Twelve intensity and intensity statistics features were extracted from the strut bloom and shadow region of each strut candidate. Then a bagged decision trees classifier was trained on manually labeled struts and was used to identify true stent struts from the candidates. With a machine learning approach, we avoided manually developed heuristics in traditional image processing methods. Instead, we made use of a large number of manually analyzed stent pullbacks to build a robust algorithm. Sensitivity and precision of this detection algorithm were greater than 90%.

### Stent contour estimation

A good estimation of the stent contour is needed for quantifying tissue coverage area and malapposition area. The stent contour is typically a manually drawn closed contour connecting stent struts in a smooth cylindrical fashion creating the stent “envelope”. We created the stent contour using a periodic cubic spline (PCS) on strut locations^25^. Recently, we added improvements to our previous algorithm giving more realistic contours. First, the stent contour given by PCS was not always smooth when struts were not uniformly distributed around the image frame. Instead of using PCS to build the final stent contour, we used the contour given by PCS to estimate the center of the stent contour. The final stent contour was then obtained by interpolating new radii lengths from strut radii as measured from the stent center. Second, when no strut was present in a quadrant of the frame, strut locations in previous and next frames were used to build the stent contour. This strategy is commonly used in manual stent analysis. When there was no strut in more than half of a frame, the stent contour was not estimated and the frame was excluded from area quantification.

### Stent strut classification

We utilized a machine learning approach to classify struts as either covered or uncovered^27^. This is the most challenging task in stent analysis when tissue coverage is thin. As reported previously^41,42^, a strut is considered as covered when it satisfies two criteria: (1) There appears to be a smooth layer of tissue covering the luminal side. (2) The strut-tissue boundary in the angular direction is continuous and homogeneous. Based on these two criteria, we derived 21 image features from 6 pixel patches surrounding the detected strut center^27^. Eighty manually labeled pullbacks from a previous study, analyzed by other analysts were used to train a support vector machine (SVM) classifier. We employed a radial basis function (RBF) kernel in SVM, which allowed the classifier to learn complicated, non-linear relationship between features. The performance of this algorithm was evaluated by 8-fold cross validation on the 80 pullbacks, giving 94% sensitivity and 90% specificity for identifying uncovered struts.

**Figure 3:**
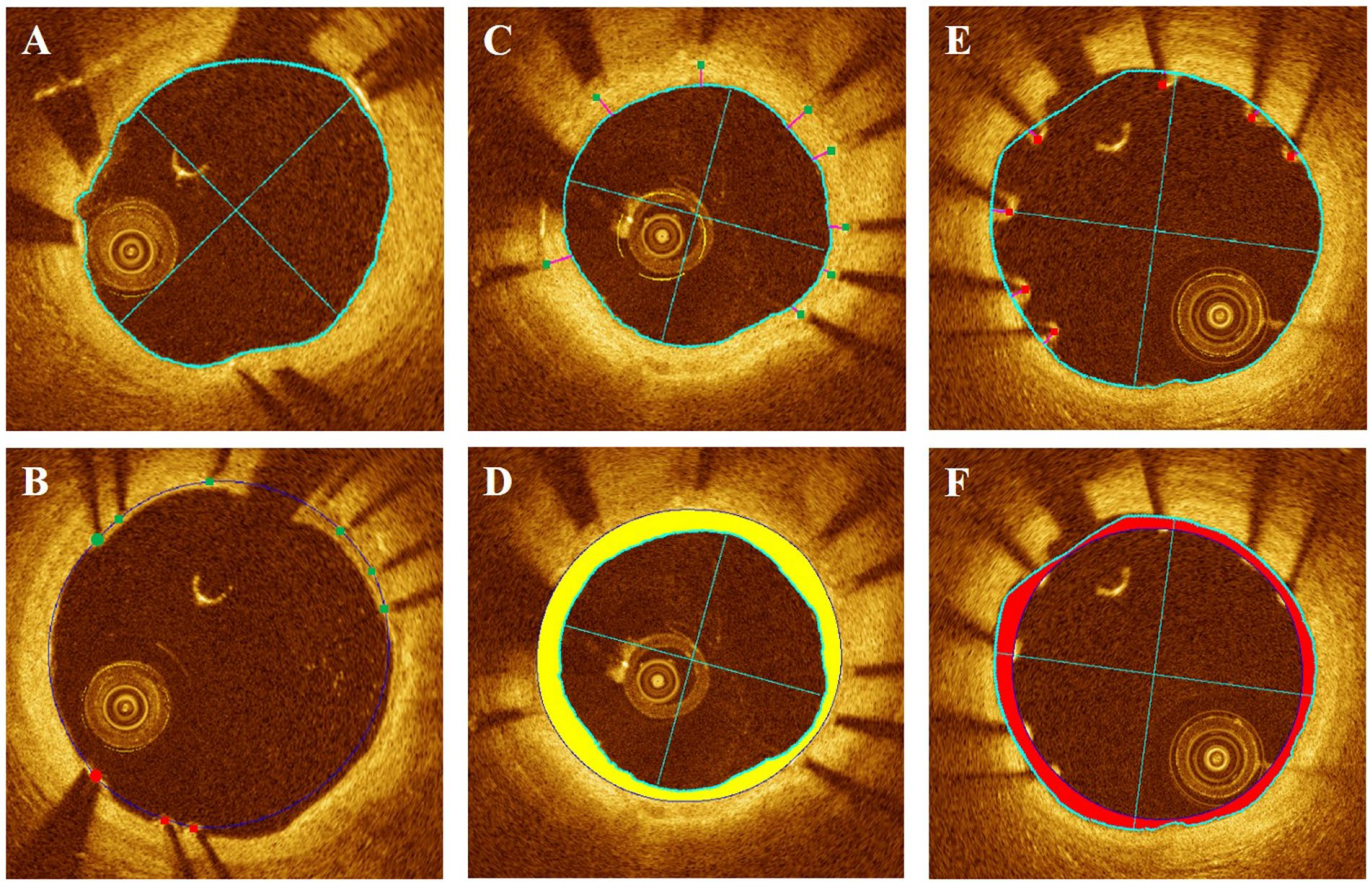
Stent analysis image output of OCTivat-Stent. (**A**) Automatic lumen detection result, lumen tracing, major and minor axes are shown in cyan. (**B**) Detected stent struts are automatically classified as covered (green) versus uncovered (red). Low-confidence predictions are highlighted in bigger dots to draw special attention. Stent contour (blue) is estimated using detected strut locations. (**C**) Strut-level tissue coverage thickness measurement (magenta). (**D**) Frame-level neointima hyperplasia area (yellow) quantification for the cross section in panel (C). (**E**) Strut-level malapposition distance measurement. (**F**) Frame-level malapposition area (red) quantification for the cross section in panel (E).

We utilized the output probability of the classifier to mark ambiguous struts for potential user editing. Since a probability of around 0.5 indicates that classification is uncertain, we provided a user specified range of values on either side of 0.5, typically 0.25–0.75 for marking struts as ambiguous. These struts were highlighted with bigger markers in the display for potential editing (see Fig. 3B in Results).

### Tissue coverage and malapposition quantification

To accurately quantify tissue coverage and malapposition, we first refined the lumen boundary based on strut classes. The lumen boundary determined from the initial DP step usually lay on the luminal side of the struts. For covered struts, this lumen boundary was preserved. For uncovered struts, the lumen boundary points in A-lines containing the strut were replaced by interpolating the lumen locations at the two strut ends in the angular direction (Fig. 3E in Results). Strut level tissue coverage thickness and malapposition distance were then measured from strut center to its closest-distance point on the refined lumen boundary. To calculate frame level tissue coverage and malapposition areas, we generated a binary stent mask using the stent contour and a lumen mask using the lumen tracing. The difference between these two masks was computed. The area with positive values in the difference mask is tissue coverage and area with negative values is malapposition. Above, for simplicity, we referred to the strut center as the bright spot in the IVOCT image. Since the reflection occurs at the front surface of the stent strut, we took into account strut thickness in measurements using the stent contour. Specifically, we expanded the stent contour by the strut thickness in the radial direction when measuring malapposition area.

## Results

### Display

Figure 3 shows output images from OCTivat-Stent. The automatically detected lumen boundary highlighted in cyan accurately followed the lumen border even though the catheter abutted the artery wall (Fig. 3A). As shown in Fig. 3B, detected stent struts were correctly classified as either covered (green) or uncovered (red). High-confidence predictions are shown in smaller squares and low-confidence predictions are shown in bigger dots. The stent contour estimated from strut locations is shown in blue. Strut-level tissue coverage thickness and tissue coverage area are shown in Fig. 3C,D, respectively. In Fig. 3E, strut-level malapposition distances are shown after lumen refinement. Malapposition area is shown in Fig. 3F.

### Lumen and stent area measurement validation

**Figure 4:**
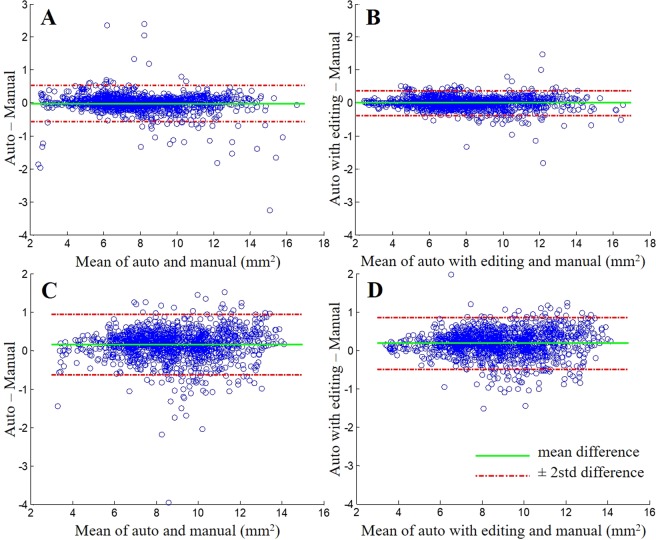
Bland-Altman plots of lumen and stent areas. (**A**) Fully automated versus manual lumen area measurements. Area differences (auto – manual) are plotted as a function of the mean of auto and manual. Any value above zero on the vertical axis indicates that the automatically determined area exceeds the manual area. (**B**) Automatic lumen area with editing versus manual. (**C**) Fully automated versus manual stent areas. (**D**) Automatic stent area with editing versus manual. The 50 pullbacks including about 1,500 cross sections were analyzed. Some outliers with fully automatic analysis (**A**,**C**) were repositioned with manual editing, reducing measurement variance (**B**,**D**).

On 50 pullbacks containing about 1,500 cross sections, we compared automated lumen and stent area measurements with and without editing to manual measurements from the commercial analysis tool (Fig. 4 and Table 1). Fully automated measurements from OCTivat-Stent correlated well with manual measurements, giving a concordance correlation coefficient (CCC) of 0.993 for lumen area and 0.979 for stent area (Table 1). CCC is superior to correlation coefficient because it assesses the equivalency of the two measurements, rather than the dependency as would be described by correlation coefficient. CCC ranges from −1 to 1, where −1 corresponds to perfect negative agreement and 1 corresponds to perfect agreement. Manual editing further improved this correlation and reduced measurement difference.

**Table 1:** Lumen and stent area measurement validation.

	Automatic VS. Manual		Automatic + Editing VS. Manual	
	Difference (mm^2^)	CCC	Difference (mm^2^)	CCC
Lumen area	−0.01 ± 0.28	0.993	−0.00 ± 0.19	0.997
Stent area	0.15 ± 0.40	0.979	0.18 ± 0.34	0.983

Figure 4 shows Bland-Altman plots of area measurements. The Bland-Altman plot, commonly used to evaluate agreement between two different methods for measuring the same variable, was generated by plotting the difference of two measurements against the mean of the two for each measured sample. In Fig. 4A, fully automated lumen area measurements were compared to corresponding manual measurements. There were errors in automated measurements giving rise to spread of data points in the vertical direction, which was further assessed with standard deviation (red lines). The mean difference (green line) was very near zero indicating no bias in the automated measurement. This analysis was repeated after manual editing of about 5–10% of image frames (Fig. 4B). Outliers were repositioned and standard deviation was reduced. After editing, we obtained a bias of 0% and a standard deviation of 2.4% of the mean (8 *mm*^2^). In Fig. 4C,D, this same analysis was applied to stent area without and with manual editing, respectively. Once again, outliers and standard deviation were reduced with editing, although the effect was not as dramatic as for lumen area. Please note the different vertical scales in Fig. 4C,D as compared to 4 A and 4B. After editing, we obtained + 2.0% bias and a 3.8% standard deviation as compared to mean stent area (8.85 *mm*^2^). As described in Discussion, the larger areas from OCTivat-Stent might be more appropriate than manual stent areas.

### Manual correction analysis

Manual correction of strut detection and classification was recorded to evaluate software performance. Automated analysis missed 11% of struts, and falsely identified 1% of struts. For covered versus uncovered strut classification, 18% uncovered struts and 1% covered struts were misclassified by the automated software, as determined by the cardiologist. For each pullback, we computed the percentage of uncovered struts before and after manual editing. The CCC between the percentages of uncovered struts before and after editing was 0.96, indicating percentages of uncovered struts given by fully automatic analysis were very close to final percentages after manual correction (see Discussion).

**Figure 5:**
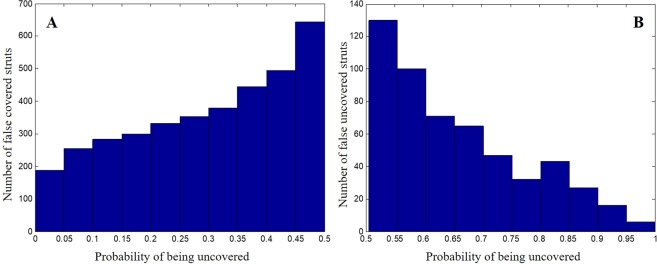
Probabilities of being uncovered for manually relabeled struts. (**A**) Probabilities for struts automatically classified as covered and manually relabeled as uncovered. (**B**) Probabilities for struts automatically classified as uncovered and manually relabeled as covered. More struts are relabeled by the cardiologist when the probability of being uncovered is close to 0.5, where the classifier has a reduced confidence in its prediction. Sixty-five percent of relabeling occurs between probabilities 0.25 and 0.75.

As the SVM classifier gave probabilistic outputs, we analyzed the probability of being uncovered for the relabeled struts (Fig. 5). When probability was close to 0.5, the classifier had reduced confidence in its prediction. The histograms were peaked towards probability of 0.5, indicating that struts tended to be relabeled when prediction confidence was low. This suggests that we can assist manual review by highlighting struts with low prediction confidence as targets for potential editing (Fig. 3B).

### Stent analysis time

**Figure 6:**
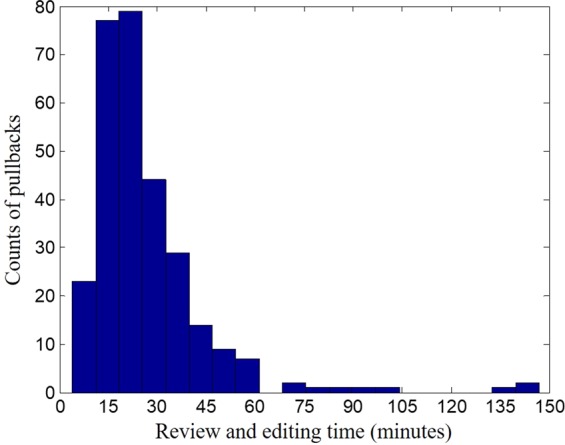
Histogram of manual review and editing time (292 pullbacks). Mean analysis time was 27 ± 18 minutes. About 3% pullbacks with poor quality required more than an hour to edit. Most pullbacks (90%) were analyzed within 45 minutes, more than 70% were analyzed within 30 minutes, and about 20% were analyzed within 15 minutes.

The amount of time needed to analyze a stent pullback is an important metric for software usability. With our non-optimized implementation in Matlab (MathWorks Inc., Natick, MA, USA), the automatic analysis time per pullback was less than 30 minutes. The only user intervention was about 5 minutes for setup. The software then ran in batch mode without user observation. Manual review and editing time per pullback is a more important metric. Figure 6 is a histogram of recorded analysis time on 292 pullbacks. Analysis time per pullback ranged from 10 minutes to 2.5 hours, 27 ± 18 (mean ± σ) minutes. Very few pullbacks (<3%) with poor image quality due to the presence of blood/thrombus took more than an hour to analyze. Most pullbacks (90%) were analyzed within 45 minutes, a significant reduction compared to 6~12 hours needed for fully manual analysis of every stent frame.

### Inter-observer variability analysis

**Figure 7:**
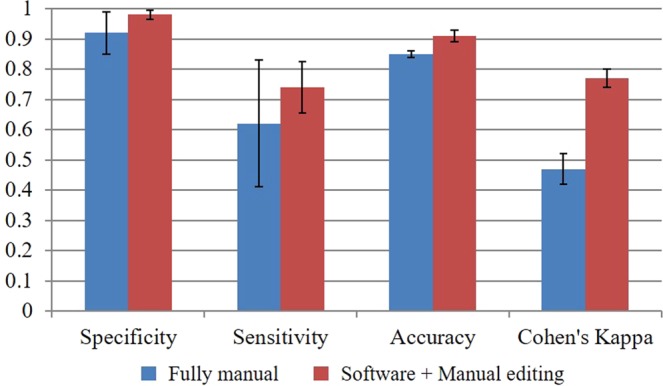
Inter-observer strut classification agreement with and without software assistance. Statistics were calculated from 3 analysts as describe in the text. As compared to fully manual analysis, inter-observer variability was reduced when analysts used OCTivat-Stent with manual review and editing. Improvements were: specificity (6%), sensitivity (12%), and accuracy (6%). Cohen’s Kappa coefficient improved greatly from 0.47 to 0.77.

To compare inter-observer agreement with and without software assistance, we asked three analysts to perform fully manual analysis and to use OCTivat-Stent with editing, on three pullbacks. This gave three pairings of analysis for each condition. Agreement between each pair was calculated, assuming one to be the gold standard, giving false positive, false negative, sensitivity, etc. Statistics were averaged across the three pairs. Covered versus uncovered classification results are shown in Fig. 7 with uncovered being a positive test result. With software assistance, specificity, sensitivity, and accuracy all substantially improved relative to results from a fully manual analysis (see legend), indicating greatly improved agreement between observers. A measurement of concordance, Cohen’s Kappa coefficient, was measured for each pair and averaged. Cohen’s Kappa increased from 0.47 ± 0.05 to 0.77 ± 0.03. This is a substantial improvement, as some report 0.41–0.60 as moderate agreement and >0.75 excellent agreement.

### Software performance in challenging cases

**Figure 8:**
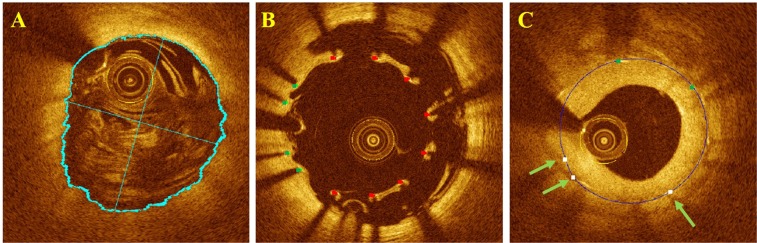
Successful automatic analysis in challenging cases. (**A**) Correct lumen boundary detection in a cross section with large amount of blood residual. (**B**) Strut detection result in an image with severe malappostion. (**C**) Stent contour estimation using strut locations in neighborhood frames (green arrows).

**Figure 9:**
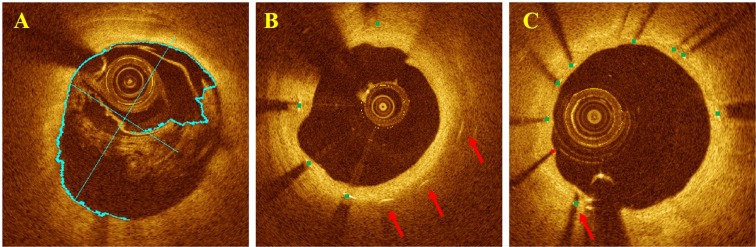
Automatic analysis errors in difficult cases requiring manual correction. (**A**) Failed lumen boundary detection in a cross section with severe blood residual. (**B**) Struts in lipid-pool missed (red arrows) due to the absence of shadow. (**C**) False positive strut detection (red arrow) due to multiple refection of strut.

**Figure 10:**
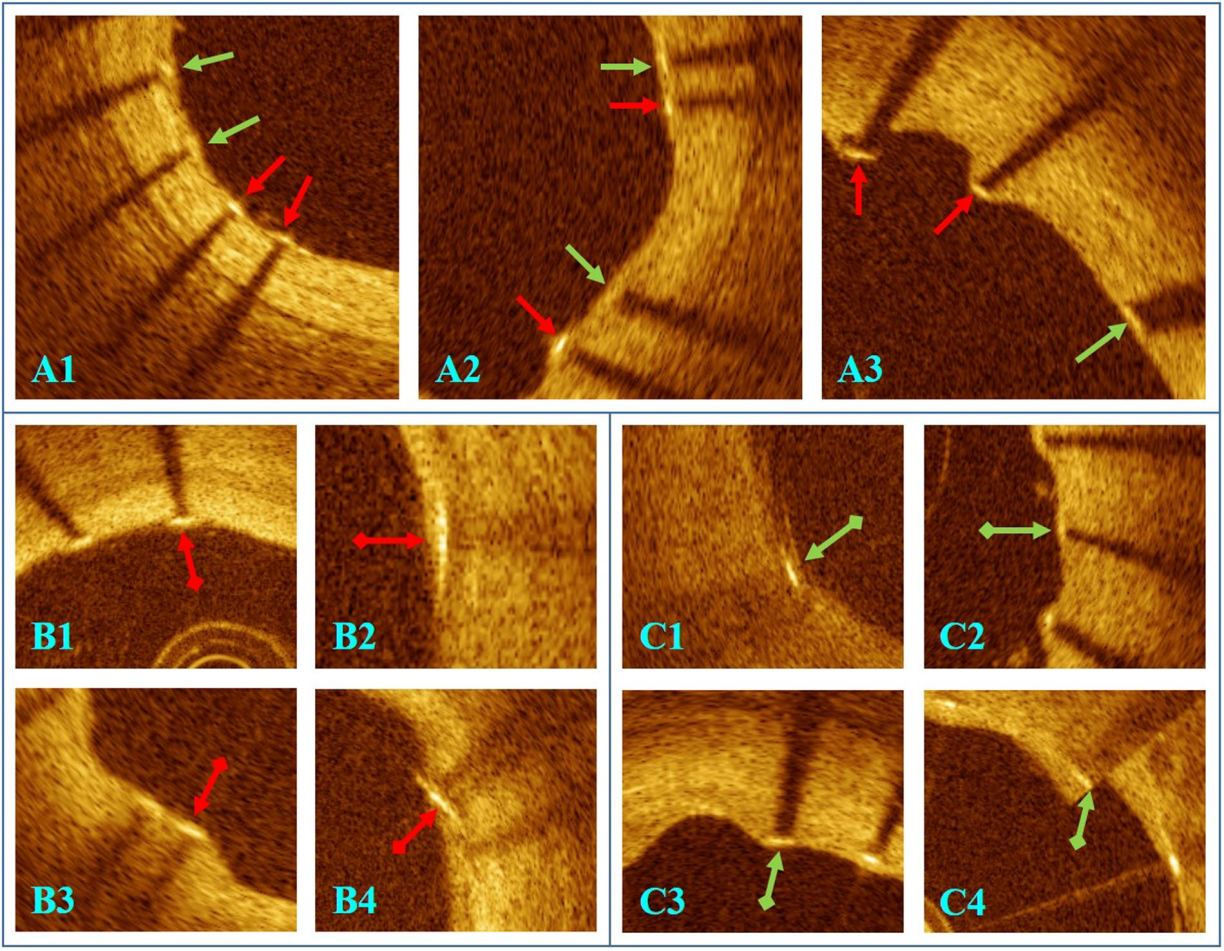
Challenges in strut classification and misclassifications requiring editing. (**A**) Algorithm correctly classified thinly covered struts (green arrows) versus uncovered struts (red arrows.) (**B**) Misclassification of uncovered struts (red arrows with square end) often happens to struts with a small part exposed to the lumen. (**C**) Misclassification of covered struts (green arrows with square end) usually happens when tissue coverage is dim due to poor image quality (**C1**), tissue coverage is very thin (**C2** and **C3**) or the strut coincides with the so-called sew-up artifact.

Our lumen segmentation algorithm was able to handle inferior image quality in most situations (Fig. 8A). Errors usually happened to images with poor quality such as severe blood residual (Fig. 9A). In Fig. 8B, we show that our software was able to capture struts in frames with severe malapposition. Struts in large lipid pool region were often missed due to the absence of shadow (Fig. 9B). False positives were rare, occasionally occurring in the presence of blood residual or multiple reflection of the strut (Fig. 9C). In Fig. 8C, we can see that, even some struts in the frame are missed, stent contour can be estimated by using strut locations in neighborhood frames. Our classifier was able to distinguish thinly covered and uncovered struts in most cases (Fig. 10A). Uncovered struts were usually misclassified as covered when only a small part of the strut was exposed to the lumen (Fig. 10B). Covered struts were often misclassified when tissue coverage was very thin or tissue coverage was dim due to poor image quality (Fig. 10C).

### Reports and visualization

**Figure 11:**
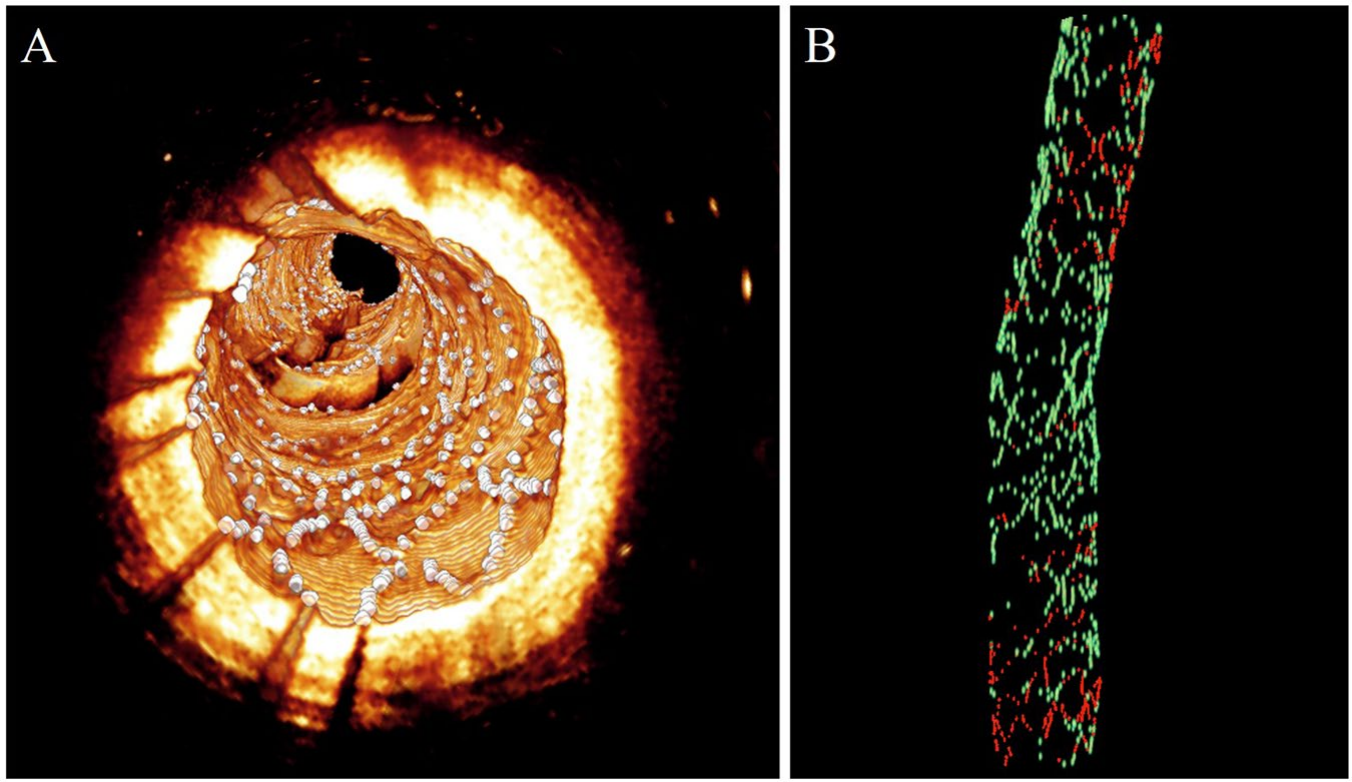
3D Visualization of analysis output of OCTivat-Stent. (**A**) Vessel wall is shown in gold and detected struts are shown in white. (**B**) Vessel is removed. Covered struts are shown in green and uncovered struts are shown in red.

**Figure 12:**
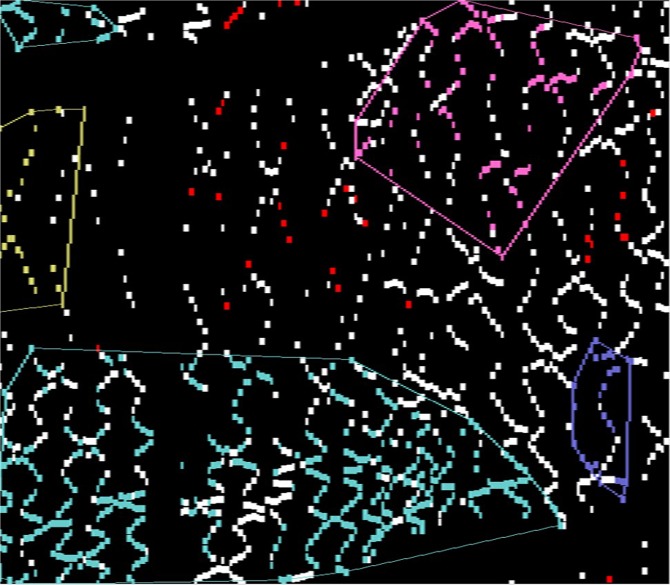
Clusters of uncovered struts. White: covered struts. Red: isolated uncovered struts. Other colors: relatively large clusters of uncovered struts.

Following lumen segmentation, strut detection, and strut classification, OCTivat-Stent computes derived measurements including lumen area and diameter, stent area, tissue coverage area, malapposition area, strut location, class (either covered, uncovered, or malapposed), percentage of uncovered struts, and coverage thickness/malapposition distance. All results are output in a comprehensive Excel report that can be easily used for various analyses, e.g. tissue coverage thickness distribution histogram, minimum & mean lumen/stent area, maximum & mean tissue coverage area, volume measurements, and maximum length of segments with uncovered/malapposed struts. In addition, OCTivat-Stent generates visualizations within the program and creates image files which can be readily visualized in 3D using a visualization program such as Amira (Thermo Fisher Scientific, Waltham, MA, USA). Examples of 3D stent reconstruction and clusters of uncovered struts are shown in Figs. 11 and 12, respectively. For clusters of uncovered struts, we can compute statistics like number of clusters, cluster area, and percentage of coverage of each cluster.

## Discussion and Future Work

We developed and evaluated a comprehensive software package, OCTivat-Stent, for analyzing IVOCT pullbacks of coronary stents. OCTivat-Stent performs automatic stent analysis using machine learning-based image processing algorithms. Lumen segmentation and stent strut detection are done automatically. Stent struts are classified as covered versus uncovered using a robust classifier. Strut level tissue coverage, malappostion distance, cross section level tissue coverage, and malapposition areas are quantified and recorded as software output. Convenient review and editing tools are built in the software to enable refinement of automated results.

We performed automatic stent analysis on 292 pullbacks (103 baseline and 189 follow-up pullbacks), mostly from the ROBUST study and a cardiologist reviewed and edited automatic analysis results. For lumen boundary detection, approximately 5–10% frames in a pullback needed manual editing. For strut classification, 18% uncovered struts and 1% covered struts were relabeled by the analyst. As the classifier used in this study was trained on other data labeled by another group of analysts in a previous study, the unbalanced performance on uncovered and covered struts could be caused by analysts, who have different operating points for calling a strut uncovered.

On a subset of 50 pullbacks with full manual analysis, OCTivat-Stent lumen and stent area measurements were compared to manual measurements made on the commercial software. Automatic analysis without editing correlated well with manual measurements. Results improved further after review and editing. Stent areas from OCTivat-Stent were slightly larger than those from manual measurements. This can be attributed to the fact that with manual analysis, analysts tended to mark the front surface of the strut bloom because the peak was not easily discernable, while automatic detection marked the brightest strut pixel. The latter position should be closer to the true position of the strut front surface. Stent area was measured from an envelope of the strut front surfaces.

OCTivat-Stent greatly reduced labor. It was possible to set up 50 pullbacks in the morning and began reviewing and editing on the first ones finished that afternoon. Analysis time was reduced from 6~12 hours to an average of 27 minutes, or only 5% of a typical manual analysis time (∼9 hours). Analysts were pleased with the software. They appreciated the reduced effort and thought that they would be more alert and make fewer errors using OCTivat-Stent than the manual software. Because effort is greatly reduced, it is possible that one analyst can complete an entire study with many patients. With manual analysis, it is necessary to engage multiple analysts, thereby leading to inter-analyst variability, even with the use of OCTivat-Stent.

Another advantage is that inter-analyst agreement was significantly improved (about 30%) using OCTivat-Stent with manual review and editing (Fig. 7). It is likely that intra-observer agreement would be similarly improved. Improved agreement of analysts will reduce the variance of measurements. This, in turn, will improve statistical power for trials comparing stent designs, application techniques, or treatments. Reduced inter-analyst variability will enable better longitudinal studies where pullbacks at different follow-up time points are analyzed. In addition, it will be easier to compare a new stent type to previously analyzed stent types, or compare studies analyzed at different core labs.

There are at least two reasons for the reduced variability among analysts when using OCTivat-Stent. First, the fully manual analysis is very fatiguing. Using OCTivat-Stent, analysts will be less fatigued and possibly more attentive, leading to more consistent analyses. Second, the presence of the automated result will tend to set an operating point for uncovered versus covered. When analysis results on individual struts are examined, it appears that analysts simply have different “operating points” for detecting struts and classifying them as covered versus uncovered. Even with good rule-based consensus criteria, interpretation can vary from one analyst to the next. When using OCTivat-Stent, analysts could be “pulled” to similar operating points. In addition to improving a single study, this could help unify analyses across studies and analysis groups.

Software could be further optimized. Automated analysis takes around 0.5 hour per pullback using a Matlab implementation. With algorithm optimization and implementation in a language such as C++, this time would significantly decrease. We can also implement deep learning networks. With a faster implementation, software could be used in the catheterization lab to provide live-time physician support concerning stent malapposition at implantation or strut coverage in a follow-up exam. In addition to processing time, there could be improvements to review and edit functions to require fewer, more institutive interactions and possibly reduce analysis time.

Although performance of software will depend upon case mix, we compare our results to those from previous studies. In our current study, sensitivity/specificity values for strut detection are 89%/99%, and, for strut coverage, values are 82%/99% when considering uncovered struts as the positive class. In our previous study^43^, we achieved high sensitivity/specificity values for strut detection of 90%/90% for baseline cases and 94%/86% for follow-up cases. In another previous study^27^, we achieved sensitivity/specificity 94%/90% for identifying uncovered struts. (In our current study, we include cases out to 9-months post implantation, likely giving several more difficult cases than in the previously reported studies.) Bonnema *et al*.^44^ provided sensitivities/specificities of 93%/99% and 81%/96% for strut detection and strut coverage, respectively, on a dataset of 1,461 images. Ughi *et al*.^45^ reported high Pearson’s correlation coefficients (>0.96) of stent strut apposition and strut coverage measurements between the automated and manual methods. For stent strut detection, Mandelias *et al*.^46^ presented an overall accuracy of 93.8% with sensitivity/specificity of 77.7%/96.4%, respectively. Wang *et al*.^47^ demonstrated sensitivities of 91.0%, 93.0%, and 94.0% for malapposed, apposed, and covered stent struts, respectively. Nam *et al*.^48^ reported a 96.5% positive predictive value and a 92.9% true positive rate for stent strut detection. Our performance values compare favorably with those from previous reports. But it must be emphasized that performance can vary greatly on case mix and on the way that images have been labeled by analysts.

Based on the software evaluation presented in this report, we believe that OCTivat-Stent has great potential to enable faster, better evaluations of stent designs, implantation methods, and treatments such as drug eluting balloons for in-stent restenosis. It is hoped that the software can provide stent biomarker quantification which will help to drive the field forward.

## Data Availability

The datasets analyzed during the current study are available from the corresponding author on reasonable request.

## References

[1] Bezerra HG, Costa MA, Guagliumi G, Rollins AM, Simon DI (2009). Intracoronary optical coherence tomography: a comprehensive review clinical and research applications. JACC Cardiovasc. Interv..

[2] Lim JW (2019). Effects of lowest-dose vs. highest-dose pitavastatin on coronary neointimal hyperplasia at 12-month follow-up in type 2 diabetic patients with non-ST elevation acute coronary syndrome: an optical coherence tomography analysis. Heart Vessel..

[3] Seung-Yul L (2018). Early Follow-Up Optical Coherence Tomographic Findings of Significant Drug-Eluting Stent Malapposition. Circulation: Cardiovascular Interventions.

[4] Gatto L (2018). Role of optical coherence tomography in identifying sub-optimal stent positioning and predicting major adverse cardiac events in a comparative study with angiography: a CLIO-OPCI II sub-study. Coron. Artery Dis..

[5] Suzuki Y (2008). *In vivo* comparison between optical coherence tomography and intravascular ultrasound for detecting small degrees of in-stent neointima after stent implantation. JACC Cardiovasc. Interv..

[6] Capodanno D (2009). Comparison of optical coherence tomography and intravascular ultrasound for the assessment of in-stent tissue coverage after stent implantation. EuroIntervention.

[7] Lüscher TF (2007). Drug-eluting stent and coronary thrombosis: biological mechanisms and clinical implications. Circulation.

[8] Pfisterer Matthias E (2008). Late Stent Thrombosis After Drug-Eluting Stent Implantation for Acute Myocardial Infarction. Circulation.

[9] Finn Aloke V (2007). Vascular Responses to Drug Eluting Stents. Arteriosclerosis, Thrombosis, Vasc. Biol..

[10] Guagliumi G (2010). Optical Coherence Tomography Assessment of *In Vivo* Vascular Response After Implantation of Overlapping Bare-Metal and Drug-Eluting Stents. JACC: Cardiovascular Interventions.

[11] Tahara S, Chamié D, Baibars M, Alraies C, Costa M (2011). Optical coherence tomography endpoints in stent clinical investigations: strut coverage. Int. J. Cardiovasc. Imaging.

[12] Giulio G (2010). Strut Coverage and Vessel Wall Response to a New-Generation Paclitaxel-Eluting Stent With an Ultrathin Biodegradable Abluminal Polymer. Circulation: Cardiovascular Interventions.

[13] Guagliumi G (2011). Strut coverage and late malapposition with paclitaxel-eluting stents compared with bare metal stents in acute myocardial infarction: optical coherence tomography substudy of the Harmonizing Outcomes with Revascularization and Stents in Acute Myocardial Infarction (HORIZONS-AMI) Trial. Circulation.

[14] Guagliumi G (2010). Strut Coverage and Vessel Wall Response to Zotarolimus-Eluting and Bare-Metal Stents Implanted in Patients With ST-Segment Elevation Myocardial Infarction: The OCTAMI (Optical Coherence Tomography in Acute Myocardial Infarction) Study. JACC: Cardiovascular Interventions.

[15] Kauffmann C, Motreff P, Sarry L (2010). *In Vivo* Supervised Analysis of Stent Reendothelialization From Optical Coherence Tomography. IEEE Trans. Med. Imaging.

[16] Bruining, N., Sihan, K., Ligthart, J., Winter, S. de & Regar, E. Automated three-dimensional detection of intracoronary stent struts in optical coherence tomography images. In *2011 Computing in Cardiology* 221–224 (2011).

[17] Adriaenssens T (2014). Automated detection and quantification of clusters of malapposed and uncovered intracoronary stent struts assessed with optical coherence tomography. Int. J. Cardiovasc. Imaging.

[18] Xu C, Schmitt JM, Akasaka T, Kubo T, Huang K (2011). Automatic detection of stent struts with thick neointimal growth in intravascular optical coherence tomography image sequences. Phys. Med. Biol..

[19] Ughi GJ (2012). Automatic segmentation of *in-vivo* intra-coronary optical coherence tomography images to assess stent strut apposition and coverage. Int. J. Cardiovasc. Imaging.

[20] Ughi GJ (2014). Automatic assessment of stent neointimal coverage by intravascular optical coherence tomography. Eur. Heart J. - Cardiovascular Imaging.

[21] Bonnema GT, Cardinal KO, Williams SK, Barton JK (2008). An automatic algorithm for detecting stent endothelialization from volumetric optical coherence tomography datasets. Phys. Med. Biol..

[22] Gurmeric S, Isguder GG, Carlier S, Unal G (2009). A new 3-D automated computational method to evaluate in-stent neointimal hyperplasia in *in-vivo* intravascular optical coherence tomography pullbacks. Med. Image Comput. Comput Assist. Interv..

[23] Wang A (2013). Automatic stent strut detection in intravascular optical coherence tomographic pullback runs. Int. J. Cardiovasc. Imaging.

[24] Tsantis S (2012). Automatic vessel lumen segmentation and stent strut detection in intravascular optical coherence tomography. Med. Phys..

[25] Lu H (2012). Automatic stent detection in intravascular OCT images using bagged decision trees. Biomed. Opt. Express, BOE.

[26] Nam HS (2016). Automated detection of vessel lumen and stent struts in intravascular optical coherence tomography to evaluate stent apposition and neointimal coverage. Med. Phys..

[27] Lu H (2019). Automated stent coverage analysis in intravascular OCT (IVOCT) image volumes using a support vector machine and mesh growing. Biomed. Opt. Express, BOE.

[28] Wang Z (2010). Semiautomatic segmentation and quantification of calcified plaques in intracoronary optical coherence tomography images. J. Biomed. Opt..

[29] Wang Z (2012). Volumetric quantification of fibrous caps using intravascular optical coherence tomography. Biomed. Opt. Express.

[30] Shalev, R., Bezerra, H. G., Ray, S., Prabhu, D. & Wilson, D. L. Classification of calcium in intravascular OCT images for the purpose of intervention planning. *Proc SPIE Int Soc Opt Eng* **9786** (2016). 10.1117/12.2216315 PMC5873316 29606786

[31] Kolluru, C., Prabhu, D., Gharaibeh, Y., Wu, H. & Wilson, D. L. Voxel-based plaque classification in coronary intravascular optical coherence tomography images using decision trees. *Proc SPIE Int Soc Opt Eng* **10575** (2018). 10.1117/12.2293226 PMC5860831 29568146

[32] Kolluru C (2018). Deep neural networks for A-line-based plaque classification in coronary intravascular optical coherence tomography images. J. Med. Imaging.

[33] Prabhu, D. *et al*. 3D registration of intravascular optical coherence tomography and cryo-image volumes for microscopic-resolution validation. *Proc SPIE Int Soc Opt Eng* **9788** (2016). 10.1117/12.2217537 PMC4859892 27162417

[34] Shalev, R. *et al*. Intravascular optical coherence tomography image analysis method. In *2015 41st Annual Northeast Biomedical Engineering Conference (NEBEC)* 1–2, doi:10.1109/NEBEC.2015.7117058(2015).

[35] Prabhu DS (2019). Automated A-line coronary plaque classification of intravascular optical coherence tomography images using handcrafted features and large datasets. JBO.

[36] Hong, L. *et al*. Automated stent coverage analysis in intravascular OCT (IVOCT) image volumes using support vector machine and mesh growing. *Biomedical Optics Express*, Submitted (2019). 10.1364/BOE.10.002809 PMC6583335 31259053

[37] Wang Z (2015). 3-D Stent Detection in Intravascular OCT Using a Bayesian Network and Graph Search. IEEE Trans. Med. Imaging.

[38] Kala P (2018). OCT guidance during stent implantation in primary PCI: A randomized multicenter study with nine months of optical coherence tomography follow-up. Int. J. Cardiology.

[39] Jakl, M. *et al*. P3176Stent malapposition is associated with unfavorable long-term outcomes in patients treated by primary coronary angioplasty: six-year follow-up of ROBUST study. *Eur Heart J* **39** (2018).

[40] Taniwaki Masanori (2016). Mechanisms of Very Late Drug-Eluting Stent Thrombosis Assessed by Optical Coherence Tomography. Circulation.

[41] Attizzani GF, Bezerra HG (2013). Contemporary assessment of stent strut coverage by OCT. Int. J. Cardiovasc. Imaging.

[42] Tearney GJ (2012). Consensus Standards for Acquisition, Measurement, and Reporting of Intravascular Optical Coherence Tomography Studies. J. Am. Coll. Cardiology.

[43] Lu H (2012). Automatic stent detection in intravascular OCT images using bagged decision trees. Biomed. Opt. Express.

[44] Bonnema GT, Cardinal KO, Williams SK, Barton JK (2008). An automatic algorithm for detecting stent endothelialization from volumetric optical coherence tomography datasets. Phys. Med. Biol..

[45] Ughi GJ (2012). Automatic segmentation of *in-vivo* intra-coronary optical coherence tomography images to assess stent strut apposition and coverage. Int. J. Cardiovasc. Imaging.

[46] Mandelias K (2013). Automatic quantitative analysis of in-stent restenosis using FD-OCT *in vivo* intra-arterial imaging. Med. Phys..

[47] Wang A (2013). Automatic stent strut detection in intravascular optical coherence tomographic pullback runs. Int. J. Cardiovasc. Imaging.

[48] Nam HS (2016). Automated detection of vessel lumen and stent struts in intravascular optical coherence tomography to evaluate stent apposition and neointimal coverage. Med. Phys..

