# Author Correction: Application and Evaluation of Highly Automated Software for Comprehensive Stent Analysis in Intravascular Optical Coherence Tomography

**DOI:** 10.1038/s41598-020-74850-y

**Published:** 2020-10-23

**Authors:** Hong Lu, Juhwan Lee, Martin Jakl, Zhao Wang, Pavel Cervinka, Hiram G. Bezerra, David L. Wilson

**Affiliations:** 1 Present Address: Microsoft, Azure Global, Cambridge, MA 02142 USA; 2 Department of Biomedical Engineering, Case Western Reserve University, Cleveland, OH 44106 USA; 3 University of Defense, Faculty of Military Health Sciences, Hradec Kralove, Czech Republic; 4 Department of Electrical Engineering and Computer Science, Research Laboratory of Electronics, Massachusetts Institute of Technology, Cambridge, MA 02139 USA; 5 Department of Cardiology, Krajska zdravotni a.s., Masaryk Hospital, UJEP Usti nad Labem, Usti nad Labem, Czech Republic; 6 Cardiovascular Imaging Core Laboratory, Harrington Heart & Vascular Institute, University Hospitals Cleveland Medical Center, Cleveland, OH 44106 USA; 7 Department of Radiology, Case Western Reserve University, Cleveland, OH 44106 USA

Correction to: *Scientific Reports* 10.1038/s41598-020-59212-y, published online 07 February 2020

The original version of this Article contained an error in Affiliation 3, which was incorrectly given as ‘First Department of Cardio-Angiology and Internal Medicine, Faculty Hospital Hradec Kralove, Hradec Kralove, Czech Republic’. The correct affiliation is listed below:

 University of Defense, Faculty of Military Health Sciences, Hradec Kralove, Czech Republic.

This error has now been corrected in the HTML and PDF versions of the Article.

